# Mechanical and phytochemical protection mechanisms of *Calligonum comosum* in arid deserts

**DOI:** 10.1371/journal.pone.0192576

**Published:** 2018-02-07

**Authors:** Sameh Soliman, Mohammad G. Mohammad, Ali A. El-Keblawy, Hany Omar, Mohamed Abouleish, Mohamed Madkour, Attiat Elnaggar, Racha M. Hosni

**Affiliations:** 1 Sharjah Institute for Medical Research, University of Sharjah, Sharjah, United Arab Emirates; 2 College of Pharmacy, University of Sharjah, Sharjah, United Arab Emirates; 3 Faculty of Pharmacy, Zagazig University, Zagazig, Egypt; 4 Department of Medical Laboratory Sciences, Collage of Health Sciences, University of Sharjah, Sharjah, United Arab Emirates; 5 Department of Applied Biology, University of Sharjah, Sharjah, United Arab Emirates; 6 Research Institutes of Science and Engineering, University of Sharjah, Sharjah, United Arab Emirates; 7 Department of Biology, Chemistry and Environmental Sciences, College of Arts and Sciences, American University of Sharjah, Sharjah, UAE; National Taiwan University, TAIWAN

## Abstract

Unlike animals, plants are sessile organisms, lacking circulating antibodies and specialized immune cells and are exposed to various harsh environmental conditions that make them at risk of being attacked by different pathogens and herbivores. Plants produce chemo-signals to respond to the surroundings and be able to distinguish between harmless and harmful signals. In this study, the production of phytochemicals as plant signaling mechanisms and their defensive roles in disease resistance and repelling herbivores are examined in *Calligonum comosum*. *C*. *comosum* is a leafless standalone perennial shrub widespread in sand dunes. The plant has the ability to survive the drastic environmental conditions of the arid/ hyperarid deserts of the Arabia. Structural anatomy and phytochemicals analyses were used to identify both mechanical and chemical defensive mechanisms in *C*. *comosum*. Microscopy-based investigations indicated that stems of this species developed hard structures in its outer layers including sclerenchyma and cluster crystals of calcium oxalate (CaOx). Sclerenchyma and CaOx are difficult to be eaten by herbivores and insects and can harm their mouthparts. On the other hand, the plant developed both short-distance (local) and long-distance (systematic over limited sphere) phytochemicals-producing cells located at its outer regions that is surrounding the inner nutrient-rich vascular system (VS). Local chemical was represented by phenolic idioblasts that were released in response to plant cutting. Systematic chemical was represented by toxic volatile oil containing ~50% benzaldehyde derivative (cuminaldehyde). The oil caused strong killing effect on both mammalian cells and microbial pathogens via either direct addition or indirect exposure to its vapor. The plants lost the oil content and allowed fungal growth once cut and dried. The localization of both defensive mechanisms to the outer region of the plant seemed to protect the inner nutrient-rich VS and hence maintained the plant survival. Surprisingly, in relation to traditional folklore use as medicine, local people use only green parts of the plant and only during the winter, where the plant found devoid of volatile oil and phenolic idioblasts. Moreover, it turns into recommendations for local people to avoid any health problems caused by the plant supply.

## Introduction

Plants developed several defense strategies to cope with a huge diversity of unfavorable biotic conditions. Even though, many microbial pathogens and herbivores are evolved to infect plants, plants are usually developing their counter-defenses to protect them from the attacking herbivores and pathogens [1]. Plants have a variety of constitutive and inducible defensive mechanisms to protect themselves against herbivores [2]. Constitutive defenses are well represented in the plant anatomical structures that can include cell barriers such as cell walls structure, waxy epidermis, and bark. Other constitutive defense structures can include intercellular idioblasts such as stone cells (sclereids), fibers and calcium oxalate. On the other hand, inducible defenses are mainly include toxic chemicals and defense-related proteins/ enzymes that are produced in response to pathogen invasion [3]. Toxic chemicals can include bitter-tasting tannins, pain-sensation prostaglandins and secondary metabolites that can be represented in three large chemical classes: terpenoids, phenolics, and alkaloids [4]. Production of toxic chemicals is usually accompanied with various deterrent effects on attacking herbivores [5]. Other responses commonly found among many plant genera include the production of volatile signals at sites of larval attacks by attracting insect predators such as wasps and mites [6]. Furthermore, specific pathogens can target the nutrient-rich vascular system (VS) that transports nutrient, sugars, water and minerals throughout the plant tissues and hence can cause plant death.

VS-invading pathogens can cause serious plant diseases that can wipe out entire plant species. Pathogens attacking the plant VS occur worldwide and affect annual crops as well as woody perennial plants [7–9]. Pathogens can enter their plant hosts through wounds or cracks present on the plant outer surface [10, 11]. Others can enter leaves via natural openings such as stomata and stay dormant until suitable condition of invasion, such as the bacterial leaf blight pathogen of rice, *Xanthomonas oryzae* [12]. Furthermore, some pathogens are delivered directly to the VS by insects, such as *Xylella fastidiosa* bacteria or by bark beetles, such as *Ophiostoma* fungi [13, 14]. Regardless of the mechanism used by pathogens to enter their host inner tissues, they subsequently colonize the nutrient-rich vascular system where they proliferate, consume plant nutrients and hence cause plant death [15].

*Calligonum* L. (*Polygonaceae*) genus consisted of about 80 species distributed in Southern Europe, Northern Africa and Western Asia [16]. Two species are present in the UAE; *C*. *crinitum* and *C*. *comosum*. *C*. *comosum* inhabits much of the North African deserts, the desert sands of the Middle East, Pakistan and both Central and Eastern Arabia [17]. This species is ecologically important in fixing the mobile sand, preventing the erosion, and improving the soil organic content [18]. In order to survive the arid desert conditions, plants should adopt several morphological, anatomical, physiological and biochemical conditions. For example, *C*. *comosum* produces wood of higher density, with narrow, thick-walled vessels and long fibers in the narrow growth rings of stems that have significant smaller diameter under drought conditions [19]. These strong anatomical structures are required to face strong wind and sand movement. However, to maintain its survival, it does require other mechanisms for its protections from pathogens and herbivores. In this study, both structural and phytochemical protection mechanisms were identified in *C*. *comosum*.

The *Calligonum* example represents a close examination of plant anatomy and its major phytochemicals that contribute ecologically to the plant defense and disease resistance and hence long survival.

Several species of the *Polygonaceae* family including *Calligonum* are good sources of bioactive phytochemicals with medicinal properties [20]. The production of phytochemicals as plant signaling mechanisms can help plants defend pathogens and repel herbivores [5]. It has been reported that *C*. *comosum* produces several phytochemicals, such as alkaloids, phenolics, flavonoids and essential oil [21].

Dhief et al. (2011) quantified the essential oils of three wild species of *Calligonum* (*C*. *azel*, *C*. *arich* and *C*. *comosum*) grow in the Tunisian desert and identified 110 compounds, which accounted for 94.0–99.7% of the total composition of their essential oils. The major identified components of essential oils were viridiflorol (9.6%) in *C*. *azel*, hexadecanoic acid (20.1%) in *C*. *arich* and 9-octadecenoic acid (19.8%) in *C*. *comosum* [22]. In addition, Masoum et al (2013) analyzed the volatile components of *C*. *comosum* by gas chromatography-mass spectrometry (GC-MS) and identified 105 components with a concentration greater than 0.01%. Alfa-terpineol, dodecanal, benzyl benzoate and geranyl acetone were identified as major components [23]. As the yield and types of volatile oil differ according to the climate, it is important to assess the volatile oils under the hot arid climate of the UAE to understand their protective role in defense against natural enemies.

The spatial distribution of *C*. *comosum* is clumped; i.e., plants prefer to grow close to each other in small patches [18]. We assume that the produced volatile oils are magnified when *C*. *comosum* plants grow in groups and these oils may play a role in protecting the plants from the attack of local insects and pathogens. Assessing the yield and biological activity of the volatile compounds could help in understating their role as a defensive mechanism. In addition, local people use the green leaves in the preparation of different types of foods. Consequently, assessing the biological activity of the phytochemicals would help in evaluating their effect on human health too.

## Material and methods

### Light microscopy

Plant stem pieces were subjected to very thin, hand sectioning followed by either not stained or stained for 2 min with I_2_/ KI solution for detection of starch or aqueous FeCl_3_ solution for detection of phenolic idioblasts. Only thin sections were collected and fixed on microscopic slides and checked under light microscopy using both magnification power 10 and 40. On the other hand, few sections were heated in water on microscopic slides at 50°C for ~3 min followed by cooling and fixation.

### Plant extraction

*C*. *comosum* plant materials were collected from the desert of Sharjah Emirate, UAE, identified by Prof. Ali El-Keblawy from the department of Applied Biology, UOS, UAE and plant specimens were deposited at the University Herbarium. There are no specific permissions required for collecting plant samples from open desert in the UAE for scientific purposes. Furthermore, the collected plant samples for the field studies are not classified as endangered species and not under protection protocol.

Two hundred grams plant aerial parts (equal to half the weight of a wildly-growing plant) were powdered and extracted three times with either water, 99% ethanol, ethyl acetate or hexane. Briefly, the powdered plant material was covered with the solvent and hand shake for 10–15 min, followed by filtration and the filtrated was separated. The residue was re-extracted by shaking for 10 min and left overnight, followed by filtration and the filtrate was collected. The residue was further extracted for the third time by shaking for 10–15 min followed by filtration. All filtrates from the three extractions were combined and evaporated separately till dryness. An amount of the residue equal to those extracted from 20 gm plant material (20 gm assumed to be the average amount bite by an animal [24]) was dissolved in DMSO prior to testing. In a separate experiment, plant material was extracted with ethanol and part of the residual extract from ethanol was fractionated by ethyl acetate, and hexane. All ethanol, ethyl acetate, and hexane extracts were also tested.

### Volatile oil extraction

Two hundred grams air-dried plant aerial parts were subjected to hydro-steam distillation [25] for 3 hrs. The collected oil was dried over anhydrous sodium sulphate and then stored at 4°C in sealed vials prior to GC-MS analysis and testing its activity. Similar to plant extract, an amount of the oil equal to those distillated from 20 gm plant material was dissolved in DMSO and used to test their activities.

### Studying the antimicrobial activities of plant volatile oil and extracts

The antibacterial activity of *C*. *comosum* oil compared to plant water, ethanol, ethyl acetate and hexane extracts were studied against Methicillin-resistant *Staphylococcus aureus* (MRSA) and Gram-negative *E*. *coli* on Luria-Bertani (LB)-agar plates and in liquid broth media according to a modified version of Clinical and Laboratory Standards Institute (CLSI) [26]. Briefly, 0.1 mL culture containing 10^5^ CFU /mL was spread on LB-agar plates [27]. The plates were then incubated for 24 hrs at 37°C with filter discs (8 mm diameter) containing plant extracts. For the micro-dilution method, the microbial strains were incubated with the aforementioned substances into LB broth media inoculated with 10^5^ CFU/mL in 96-well microplates at 37°C for 24 hrs and the microbial growth (turbidity) was measured by microplate reader (DYNEX technologies) at OD_600_. Each test was performed in triplicate. The anti-*Candida* activities were similarly measured against *C*. *albicans* and according to a modified version of Clinical and Laboratory Standards Institute (CLSI) [26] using LB agar plates for disc diffusion assay or yeast nitrogen base (YNB) supplemented with 100 mM glucose for micro-dilution assay.

For the spore-forming fungus (*Aspergillus niger*), the antimicrobial activities were determined by using the reference procedure of the Antifungal Susceptibility Testing Subcommittee of EUCAST for spore-forming molds [28]. Briefly, flat-bottom 96 well-plates were loaded with 200 μL RPMI 1640 medium supplemented with 2% glucose and an inoculums of 2 × 10^5^ CFU/mL. Growth inhibition was determined after 24 hrs with a spectrophotometer (DYNEX technologies) at 570 nm.

Colistin, vancomycin, Ketoconazole and amphotericin B (obtained from Sigma Company) were used as positive controls against Gram-negative bacteria, Gram-positive bacteria, *Candida* and fungi and at concentrations 3, 3, 1 and 5 μg/ mL, respectively. Cultures without antimicrobials served as negative controls and all experiments were repeated in triplicate.

### In vitro testing of anti-mammalian cells activity

The anti-mammalian activity of oil was measured either by adding the oil directly to the cells followed by MTT (3-(4, 5-dimethylthiazolyl-2)-2, 5-diphenyltetrazolium bromide) assay or indirectly by exposing the cells to the oil vapor followed by MTT assay-based detection of the zone of inhibition in the wells surrounding the application point. MTT assay was performed as follows; 96-well plates were seeded with the human glioblastoma (U-87) or normal fibroblasts (F180) cells at 4000 cells/50 μL. All cell lines were purchased from Sigma-Aldrich. For direct assay, the oil was added directly to the wells and left overnight prior to MTT assay. For the indirect assay, 10μL of the essential oil placed in an empty well centered at the middle of a standard corning 96-well plate (Cat # CLS3628BC, Sigma) and sealed with Para-film. The plate was incubated alone overnight in cell culture incubator (Cat # 9040–0088, Binder) maintained at 37°C, 95% humidity and 5% CO_2_ atmosphere. A negative control experiment was employed but without addition of the oil and incubated in a separate incubator adjusted similarly. Doxorubicin 1μM was used as positive control. A negative control plate was incubated in a separate incubator. Empty wells were used as Blank. Cells viability /survival rate percentage calculated following the formula, survival rate (%) =[(*OD test* − *OD blank*) ÷ (*OD negative control* − *OD blank*)] × 100.

The method depends on the reduction activity of mitochondria to the yellow tetrazolium MTT by dehydrogenase enzyme with the production of formazan purple dye [29]. The absorbance of the reduction product was measured using spectrophotometry. The higher the absorbance and color intensity indicates the higher the activity and viability of the cells. In contrast, lower absorbance indicates cell death (apoptosis and necrosis) [30]. A 10μL MTT reagent was added to each well. The plates were incubated 2–4 hrs and the liquid was then discarded and 100μl of DMSO was added to each well until a purple color is developed for 10 min under shaking. The purple color was then measured using Multiskan Go machine (spectrophotometer) at 570nm. Each experiment was repeated 6 times.

### Gas chromatography-mass spectrometry (GC-MS)

GC-MS measurements were carried out using an Agilent Model 7683 Autosampler, 6890 Gas Chromatograph, and 5975 Inert Mass Selective Detector in the Electron Impact (EI) mode. EI energy was set to 70 eV. Separation was carried out on an Agilent HP5-MS column with dimensions 30m x 250 μm x 0.25 μm. Ultra High Purity Grade He (Airgas) was used as carrier gas with the flow set to 0.8 mL/min in constant flow mode. The initial oven temperature was set to 45°C for 1 min followed by a 30°C/min ramp to a final temperature of 300°C which was maintained for 3 min. A 3.2 min solvent delay was used. The injector temperature was set at 220°C. The MSD was set to scan the 40–1050 m/z range. Data collection and analysis were performed using MSD Enhanced Chemstation software (Agilent). Product spectra were identified by comparison of the measured fragmentation patterns to those found in the NIST 08 Mass Spectral Library.

### Statistical analysis

The data was collected and graphed using Microsoft Excel and Graph Pad 5.0 for Windows for statistical analysis. The anticancer assay and antimicrobial activities of the plant extracts were analyzed by one-way analysis of variance (ANOVA) using Dunnett’s Multiple Comparison Test. P-value <0.05 was considered as significant.

## Results and discussion

**Fig 1:**
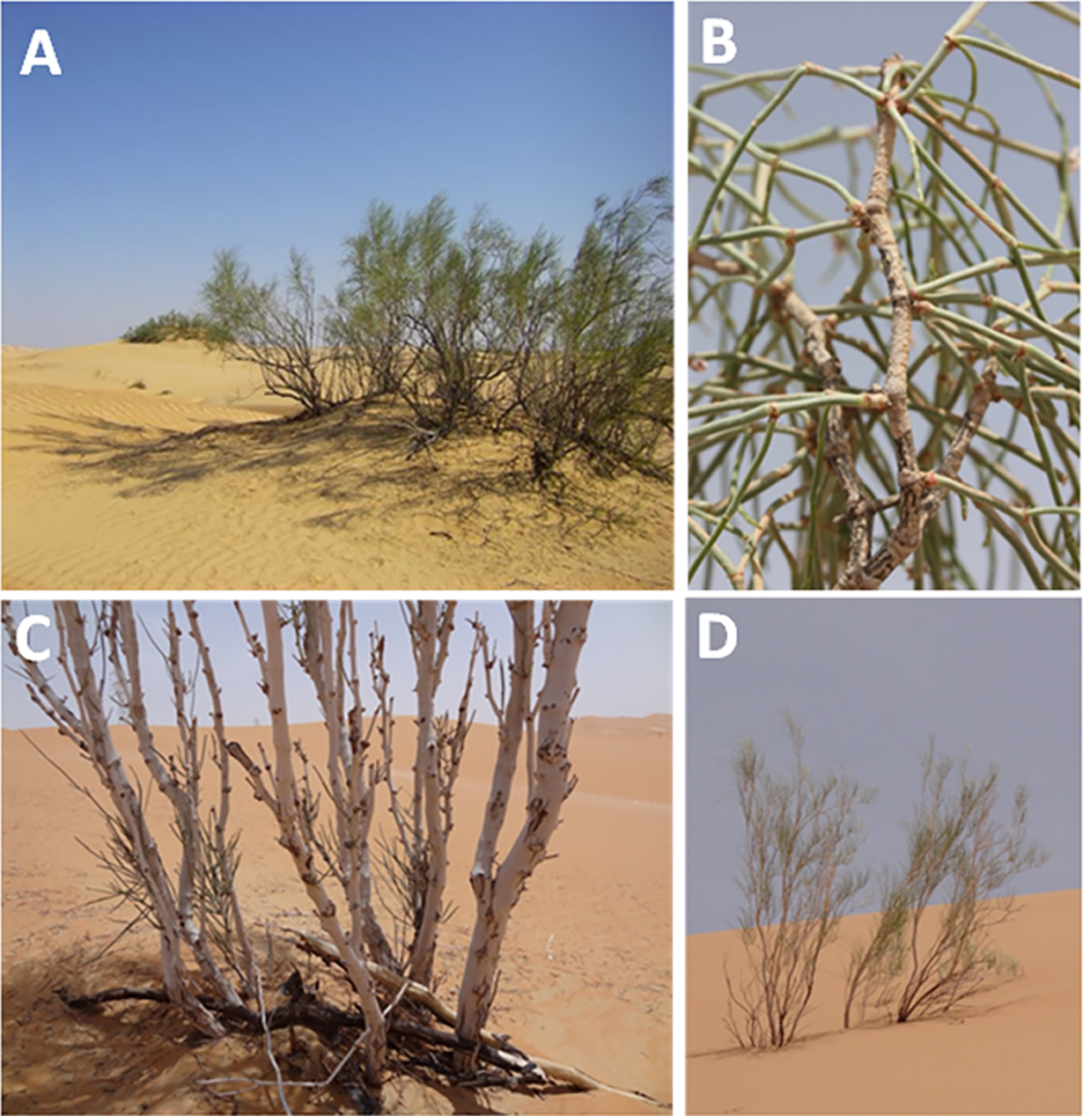
***Calligonum comosum* plant grows in the arid desert of UAE** at (A and B) winter time and (C and D) fall time [33].

*Calligonum* genus belongs to the *Polygonaceae* family, is distributed throughout Western Asia, Southern Europe and North Africa and mostly grows in course sandy deserts [31, 32]. *C*. *comosum* is an evergreen plant grows as independent small bushes in the arid desert of UAE (Fig 1). The plant shows green leafy branches (Fig 1A and 1B) during the winter which turns to scaly branches (Fig 1C and 1D) during the summer and fall. Since the plant can survive drastic environment and it can standalone in an arid land, other than drought stress, the plant exposed to pathogens/ herbivores attack. Thus the plant was investigated for the presence of specialized static plant structures and/ or movable phytochemicals mechanisms that can maintain its survival and protections from pathogen invasion and herbivores attack.

### Static mechanical mechanisms for survival of *C*. *comosum*

**Fig 2:**
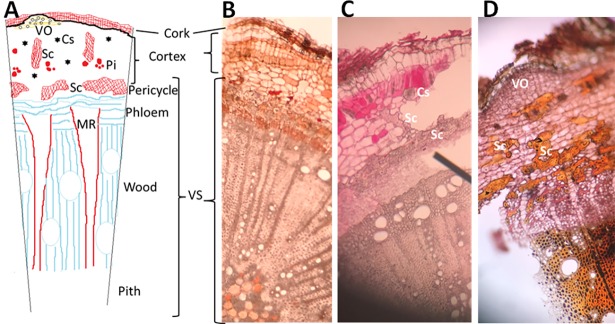
Transverse sections in *C*. *comosum* plant. (A) Diagrammatic over view of the plant section. (B) Plant section under light microscope showed all plant layers. (C and D) Close up view of the plant section under light microscope stained with neutral red and iodine solution, respectively. Cs; Cluster crystals of CaOx, MR; Medullary parenchyma rays, Pi; Phenolic idioblasts, Sc; Sclerenchyma and VO; Volatile oil.

Like most dicotyledonous plants [32, 34], a transverse section in the stem of an old *C*. *comosum* plant collected in December 2016 showed outer dead cork layer, followed by cortex and vascular system (VS). The VS clearly consisted of pericycle, phloem, xylem and pith (Fig 2A and 2B and close up view 2C and 2D).

**Fig 3:**
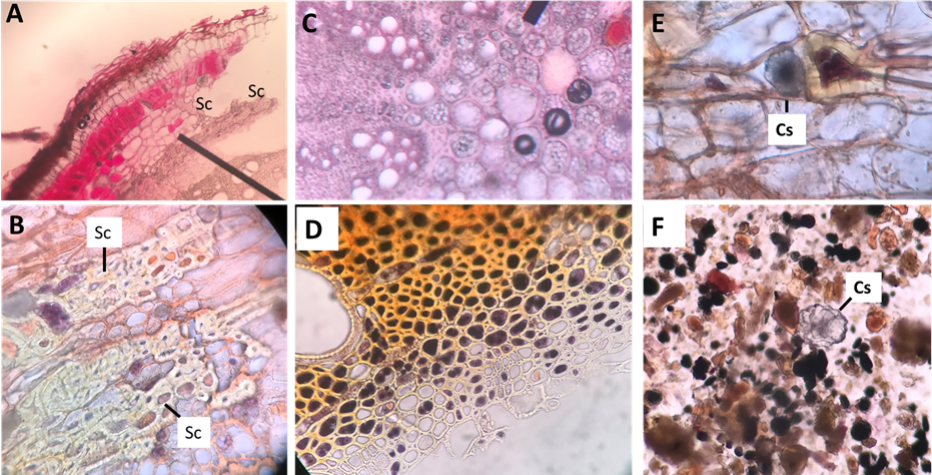
Anatomical structures of *C*. *comosum* supporting the plant hardening characteristics. (A) Transverse section of the plant indicated the location of sclerenchyma cells. (B) Close up view of the plant section indicating the sclerenchyma cells. (C) and (D) Close up view of plant sections at the vascular system showing the existence of (C) unstained and (D) iodine solution-stained starch granules. (E) and (F) Close up view of plant sections showing the presence of clusters crystals of calcium oxalate (CaOx) in (E) the outer region or in (F) the plant powder. Cs; Cluster crystals of CaOx, and Sc; Sclerenchyma.

The stem section showed the presence of an incomplete sclerenchyma (Sc) pericyclic sheath surrounding the VS (Figs 2, 3A and 3B) in addition to the existence of isolated or grouped Sc in the cortex (Figs 2, 3A and 3B). The main function of Sc is to provide the plant with hardening characteristics and hence support [35]. Mature Sc cells are known to contain secondary cell walls that are thick and hard; and hence difficult to chew and can abrasively wear down the teeth of feeding animals. Thus, Sc that forms a semi-continuous or continuous layer can protect the inner nutrient-rich vascular tissues (Fig 3C and 3D) against herbivores and insects since it is hard to be accessible [36].

The vascular tissues showed the presence of numerous starch granules (Fig 3C) that was stained black color with iodine solution (Fig 3D). Similarly, the occurrence of starch grains in the fibers of *C*. *comosum* has also been reported from the Negev desert and Saudi Arabia [19]. Such starch could be a continuous carbon source that can help the plant to survive the drought of the hyperarid Arabian deserts.

Furthermore, *Calligonum* stem cortex showed the presence of numerous cluster crystals of calcium oxalate (CaOx) (Fig 3E and 3F). The major functions of CaOx crystals formation in plants other than high-capacity calcium (Ca) regulation is the protection against herbivores [37]. The presence of CaOx in plant foods provide an important role in plant survival, but anti-nutrient and pathological impact on animal health, thus make the plant unacceptable as food [38]; the sharp cluster crystals can tear the mouthparts of insects and mammals. Aridity and ground calcium levels play important roles in the precipitation of CaOx, that might explain the presence of numerous clusters [39] in *C*. *comosum*.

### *C*. *comosum* maintains its protection and hence survival through movable and mobile phytochemicals

**Fig 4:**
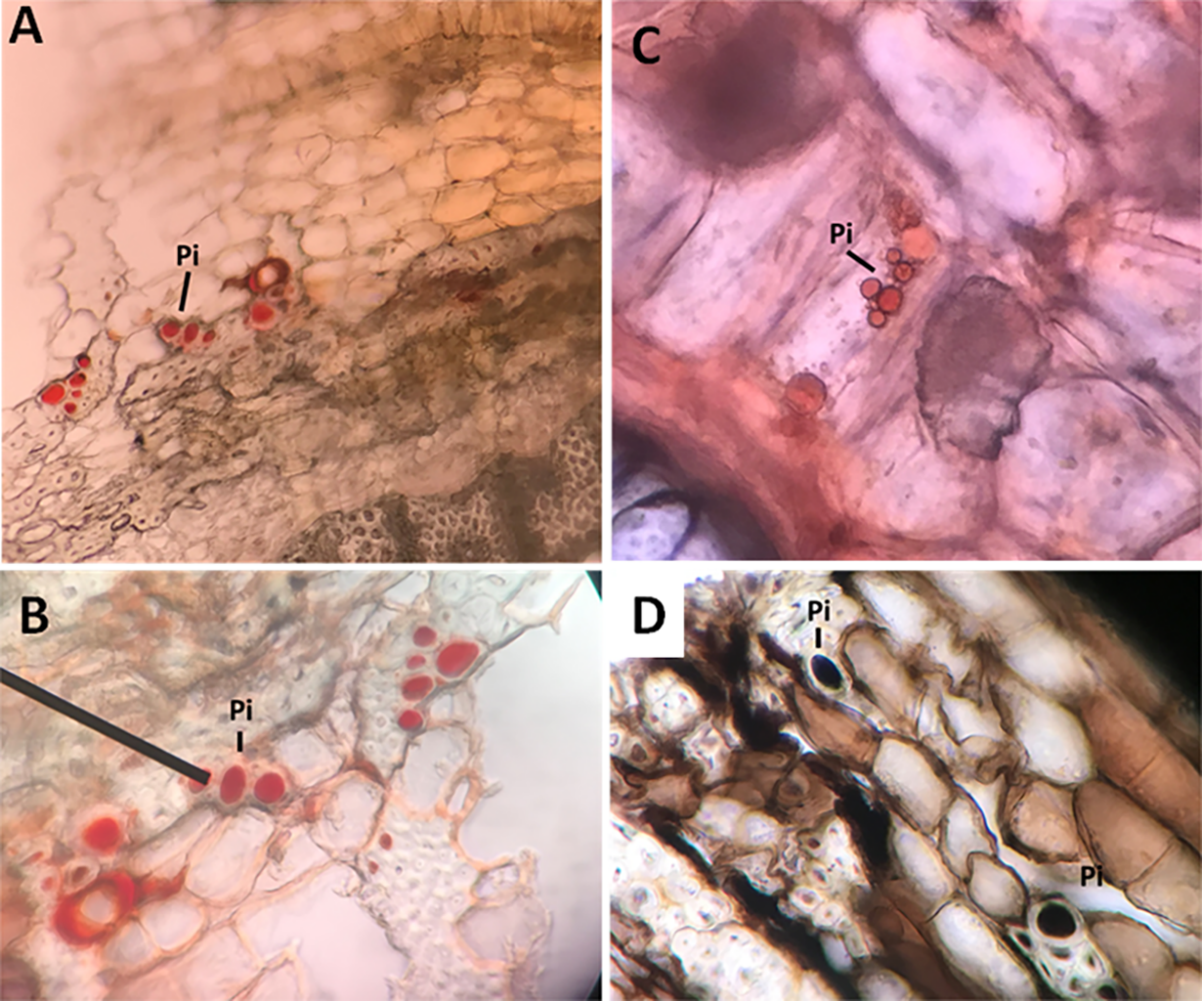
**Transverse sections of *C*. *comosum*** showing the presence of phenolic idioblasts either (A-C) unstained (red) or (D) ferric chloride solution-stained (black). Pi; Phenolic idioblasts.

**Fig 5:**
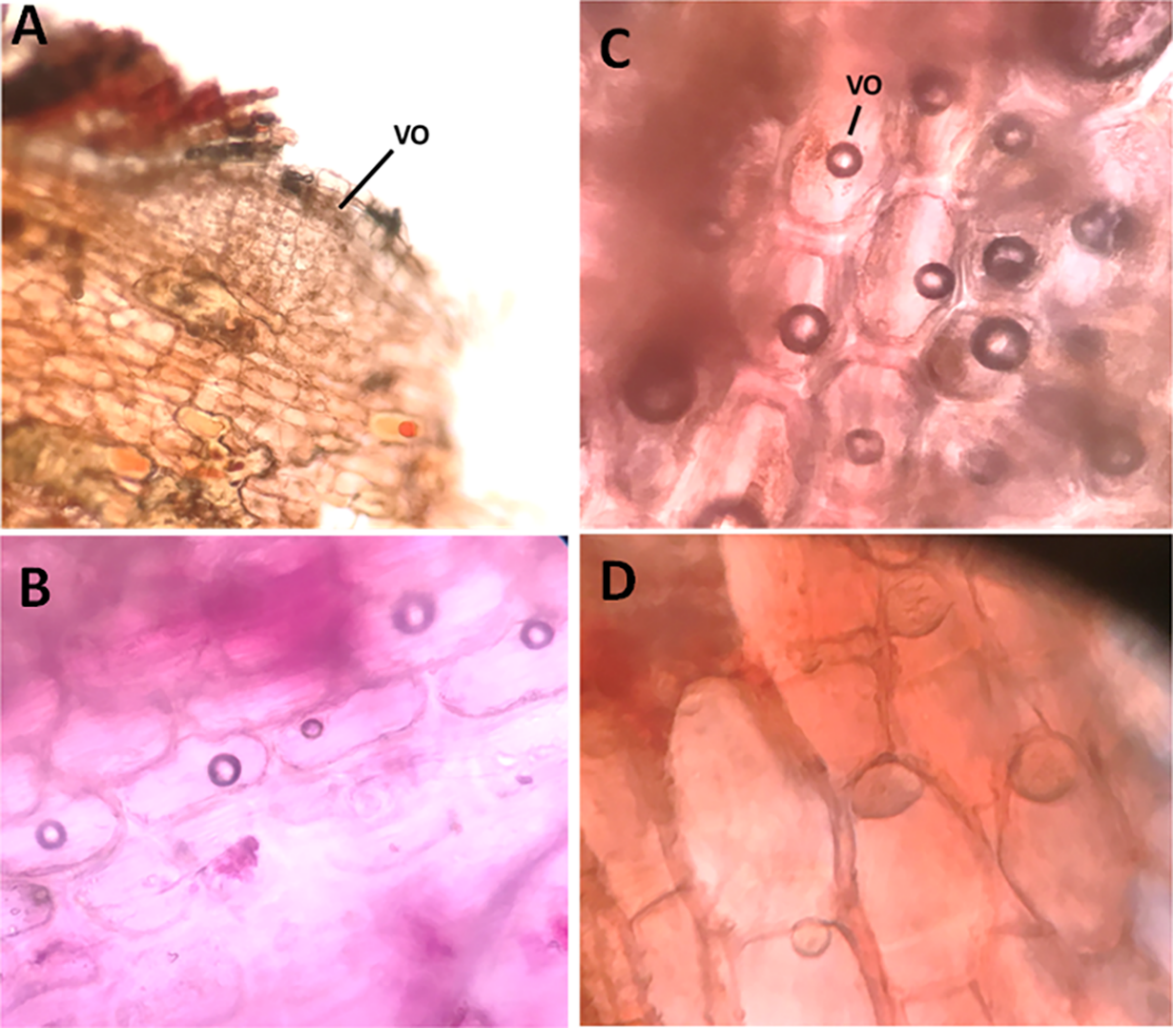
Transverse section of *C*. *comosum* plant stem showing the location of volatile oil-containing cells group at the outer surface. (A) Location of volatile oil cells. (C-D) close up view of volatile oil-containing cells. VO; Volatile oil.

Although Sc and clusters are protective structures in the plant, they cannot provide sufficient protection mechanisms; because of their occasional and static presence. Thus cannot be directed to weakened area where the pathogen and herbivores can invade. However, plants have developed movable and mobile systems (phytochemicals) designed to recognize and respond with specific defense mechanisms. The outer region of *C*. *comosum* plant showed the presence of phenolic idioblasts [34] (Fig 4A–4C) that was stained black with ferric chloride solution (Fig 4D). Similarly, the plant outer region showed the presence of groups of volatile oil-containing cells close to the plant stem surface (Fig 5).

**Fig 6:**
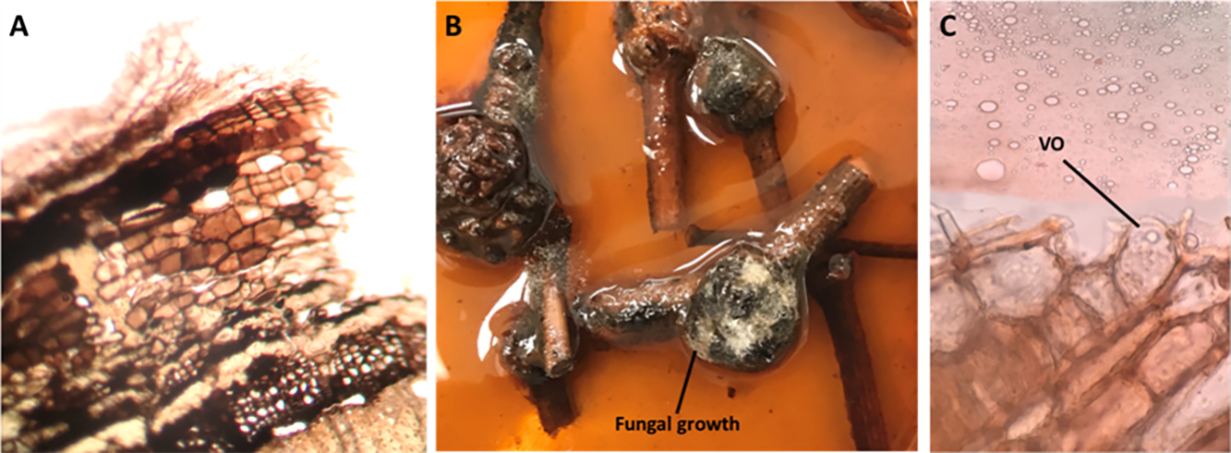
Mechanism of release of phenolics and volatile oils from old stem pieces. (A) *C*. *comosum* stem sections showed browning of outer layer when cut and left for 5 hrs. (B) Dried plant pieces left in water showing the release of phenolics outside the plant pieces and the growth of fungus. (C) Plant sections showed the release of oil once heated at 50°C. VO; Volatile oil.

Phenolic idioblasts present in the bark of woody plants can serve as constitutive anti-feeding defensive agents [40, 41]. Phenolics are known as signaling substances released in response to pathogen invasion [42, 43]. Thus cutting old *Calligonum* stem into pieces was accompanied with browning of the plant outer layers (Fig 6A). Furthermore, incubation of cut stem pieces in water for overnight was also accompanied with the release of lots of phenolics indicated by the brown coloration of water solution (Fig 6B). Badria et al (2007) identified cytotoxic phenolic compounds from *C*. *comosum* plant [21]. Phenolic extracts from *Calligonum Spp*. can include flavonoids such as kaempferol, quercetin and gallic acid that known for their antioxidant and cytotoxic effects [44, 45].

Phenolics are movable polar substances but are not mobile and hence can play a short-distance defensive role. On the other hand, volatile oils are known as less polar and mobile long-distance defensive substances that can be released easily and quickly in response to certain conditions [46, 47].

**Fig 7:**
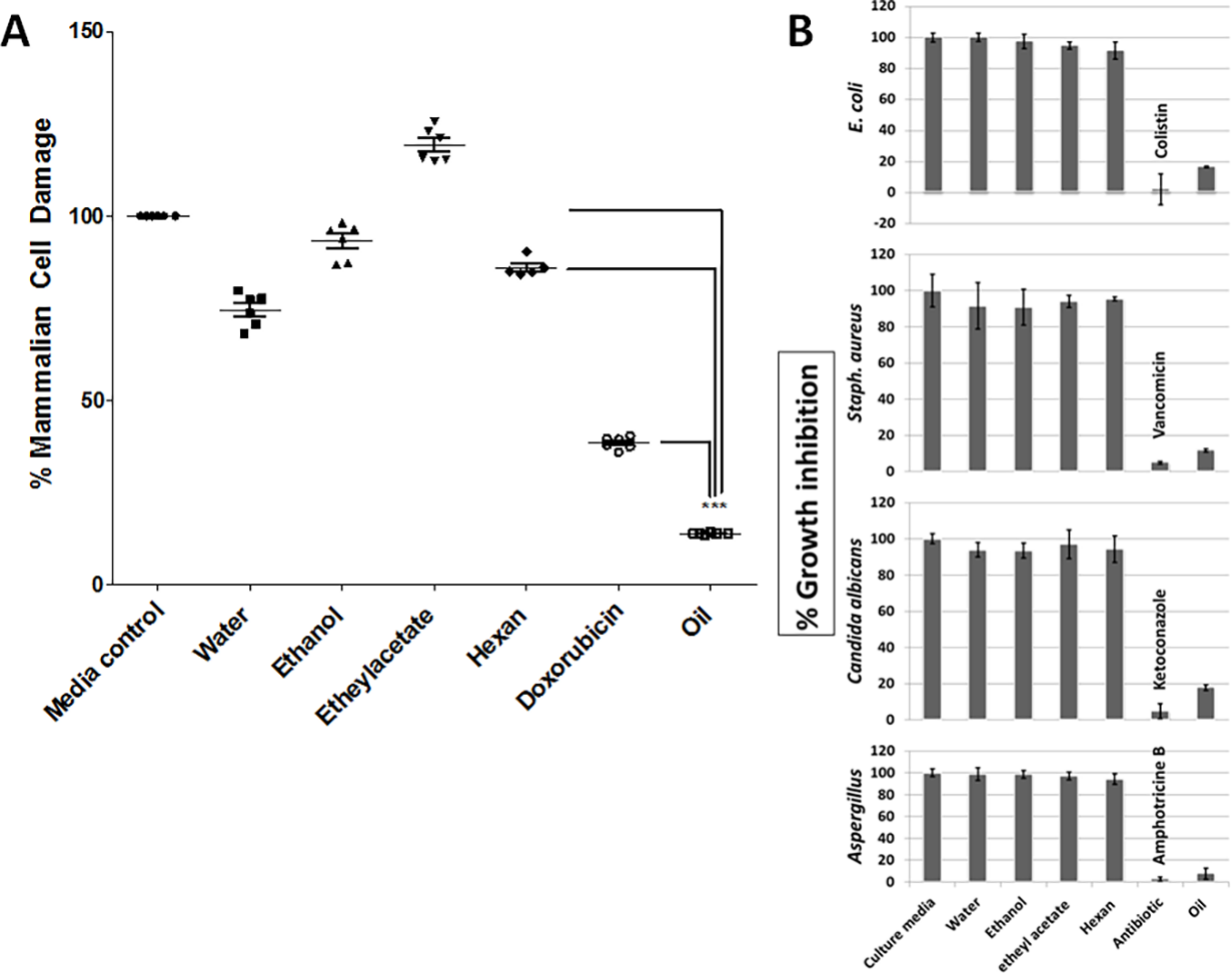
In vitro direct killing activity of plant volatile oil compared to different plant extracts. (A) MTT mammalian cell damage assay showing the effect of different plant extracts and plant volatile oil on fibroblasts cells compared to doxorubicin as positive control. As shown water extract showed slight damage to mammalian cells (due to phenolics), however oil showed significant damaging effect. (B) Antimicrobial activity of plant extracts in comparison to volatile oil and antibiotics on Gram positive *E*. *coli*, Gram negative *Staphylococcus aureus*, *Candida* yeasts and spore-forming *Asperigillus* fungus using micro-dilution assay. Colistin, vancomicin, ketoconazole and amphotricin B were used as positive controls against *E*. *coli*, *Staph*. *aureus*, *Candida* and *Aspergillus* at 3, 3, 1 and 5 μg/ mL, respectively. Each experiment was repeated six times.

**Fig 8:**
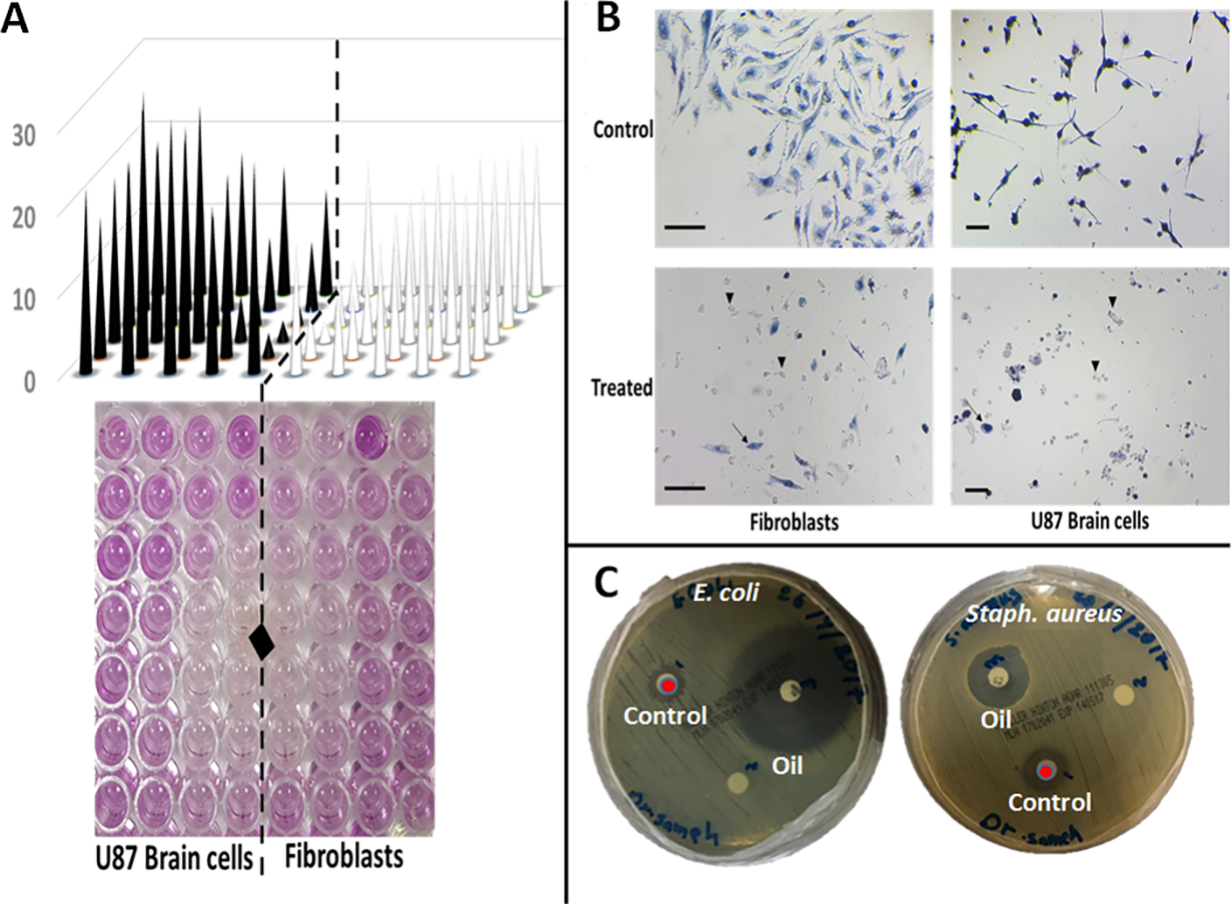
In vitro indirect activity of plant volatile oil against both mammalian cells and microbes by exposure to oil vapor. (A and B) Mammalian fibroblasts and U87 brain cells exposed to oil vapor for 24 hrs. (A) Zone of inhibition of mammalian cells when exposed to oil vapor. (B) Close up view of both cells under light microscope compared to control cells received only solvent buffer. Scale bar = 100 μm. (C) *E*. *coli* and *Staph*. *aureus* Exposed to oil vapor for 24 hrs.

Several studies have quantified and identified several types of volatile oil in *C*. *comosum* [22, 48]. The identified volatile oil in *C*. *comosum* collected from UAE was characterized by a strong musty and bed bug smell that can cause irritation of animals and made them uncomfortable. When added directly to culture media, *C*. *comosum* volatile oil showed strong killing activity against mammalian cells (Fig 7A) and different pathogens including Gram positive bacteria, Gram negative bacteria, *Candida* and spore-forming *Aspergillus* fungi compared to no or very little effect due to other tested plant extracts (Fig 7B). Furthermore, the oil showed strong indirect killing effect when its vapor exposed to mammalian human primary glioblastoma cell line (U87) compared to fibroblasts cells as control (Fig 8A and 8B) and to *E*. *coli* and *Staph*. *aureus* (Fig 8C). The selection of brain cell line based on that volatile oils are airborne molecules that carried directly to the nose by the force of inhalation and then pass to the olfactory system that initiates the smell transduction and signal regeneration in mammals including for example gazelle, camel, lion and rabbits. The regenerated signals penetrate directly to the skull and travelled to the brain where the limbic system including the hypothalamus respond by either accept or reject food [49–51]. Based on literatures using cell viability assays, an indirect inhibition of mammalian cell growth through volatile exposure has never been reported before. The cytotoxic activities of *C*. *comosum* have been reported in rat and shrimp animal models [21].

**Fig 9:**
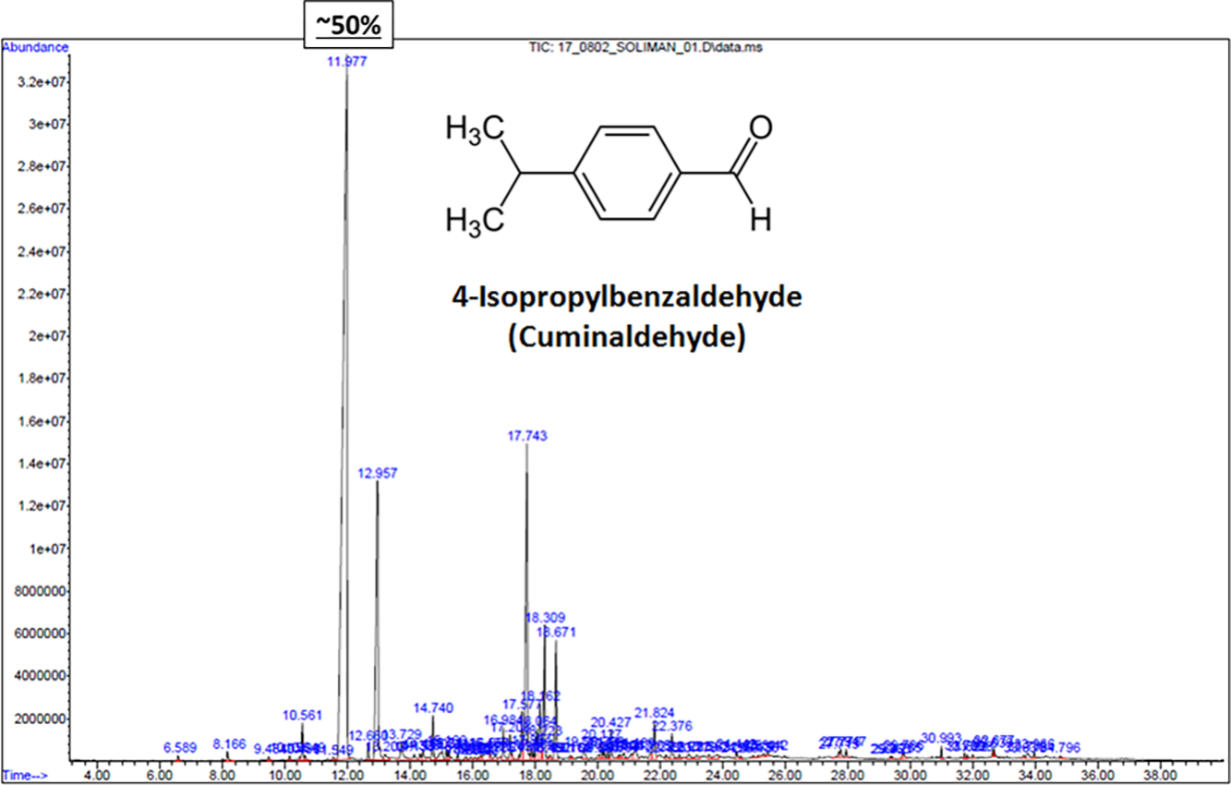
GC chromatogram of *C*. *comosum* oil showing major peaks representing ~50% cuminaldehyde at 11.9 min, ~11% carene-10-al at 12.9 min and ~10% curcumene at 17.7 min.

Analysis of *C*. *comosum* volatile oil by GC-MS indicated the presence of at least 7 major components (Table 1) including cuminaldehyde representing ~50%, carene-10-al (~11%) and curcumene (~10%) (Table 1 and Fig 9). The aforementioned major components in particular cuminaldehyde looked like unique to UAE *C*. *comosum* plant since the plant oil reported from different regions does not contain them [22, 23]. This might attributed to the difference in hyperarid hot climate of the UAE and the Mediterranean climate of other region such as Tunisia [22]; temperature can reach up to 50°C in the UAE, but rarely exceed 40°C in Tunisia. Cuminaldehyde is known as a toxic volatile substance that has a killing effect on mammalian cells, insects and microbes [52–54]. Similarly, ar-curcumene has been reported as mosquitocidal and insecticidal [55]. Mosquitoes are known as vectors for pathogens and parasites.

**Table 1:** Major identified components in *Calligonum comosum* volatile oil by GC-MS.

N	Compound	Retention time	Molecular formula	Molecular weight (g/mol)	Percentage
**1**	4-(1-methylethyl)- Benzaldehyde [Cuminaldehyde]	11.977	C10H12O	148.204	49.381%
**2**	2-Caren-10-al	12.957	C10H14O	150.220	11.314%
**3**	2-hydroxy-3-(3-methyl-2-butenyl)- 3-Cyclopenten-1-one	14.740	C10H16O2	168.235	1.011%
**4**	1,2,3,5,6,7,8,8a-octahydro-1,8a-dimethyl-7-(1-methylethenyl)-, [1S-(1α,7α,8aα)]- Naphthalene	17.575	C15H24	204.355	1.129%
**5**	1-(1,5-dimethyl-4-hexenyl)-4-methyl- Benzene [ar-Curcumene]	17.743	C15H22	202.339	9.693%
**6**	1-methyl-4-(5-methyl-1-methylene-4-hexenyl)-, (S)- Cyclohexene	18.309	C15H24	204.355	3.229%
**7**	3-(1,5-dimethyl-4-hexenyl)-6-methylene-, [S-(R*,S*)]- Cyclohexene	18.671	C15H24	204.355	2.883%

The transverse section of plant stem showed that the volatile oil is distributed in a group of cells that are exposed directly to the surface (Fig 5A). Exposure of plant stem sections for heating on a microscopic slide at ~50°C to mimic the original UAE climate allowed the release of oil outside the plant sections (Fig 6C). Furthermore, the hydrophobic nature and the surface exposure of oil can strengthen the protection effect of the oil by providing a shell that prevented the water collection on the stem surface; and hence protected against fungal pathogens that require standing water for spore germination [56, 57]. Placing dried cut plant stems devoid of oil in water allowed fungal growth (Fig 6B). This result emphasizes the defensive role of the volatile oil under the hot arid conditions of the UAE.

The distribution of *C*. *comosum* in the UAE is restricted to small patches on the coarse sandy dunes [18]. The growing of the individuals of this species close to each other in small patches indicates that the produced volatile oils might play a role in protecting the plants from pathogens, insects and herbivores. The amount of volatile oil that is produced from several plants growing in a specific patch should be high enough to deter natural enemies of *C*. *comosum*.

**Fig 10:**
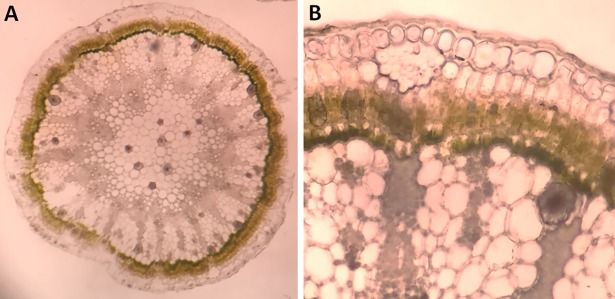
**Transverse section of *C*. *comosum*** (A) green branches collected in March and its (B) close up view under light microscope.

*C*. *comosum* is a small leafless shrub, which has a reputation in UAE folklore medicine as a stimulant and astringent, under the local names “ghardaq”, “rusah” or “arta” [58]. It has been reported to have anti-ulcer and anti-inflammatory activities [59, 60]. Surprisingly, local population use only the green parts of the plant and only during the months of February and March [61]; when, the plant is still green and devoid of toxic volatile oil or phenolic idioblasts measured by steam distillation or solvent extraction, respectively. Moreover, the shrub has small leaves that are invisible and reduced to scales during the end of the fall season where the oil and idioblasts are concentrated in the old stems. Compared to old stem, sections of green plant parts showed no phenolic idioblasts or volatile oil-containing cells (Fig 10A and 10B); indicating the safe use of the green parts by local peoples.

## Conclusions

Investigating the mechanisms used by an arid plant such as *C*. *comosum* for its protection and survival can help both ecological and medical scientific communities. From the ecological point of view, plants are evolved with their structural and/ or chemical protection mechanisms that help their survival in harsh desert conditions of the UAE, where temperatures are very high and rainfall is a rare event. UAE *C*. *comosum* is considered as a valuable example that can add to the ecological protection behaviors of plants in general and specifically for those from arid environment. Furthermore, such information may help to design a system that allows the growth of our valuable crops under arid and pathological conditions. From the medicinal point of view, understanding the phytochemical contents in plants and their structural components can be essential in order to avoid toxic food supply from causing any health problems and gives recommendations to local people.
